# *Ces* locus embedded proteins control the non-ribosomal synthesis of the cereulide toxin in emetic *Bacillus cereus* on multiple levels

**DOI:** 10.3389/fmicb.2015.01101

**Published:** 2015-10-13

**Authors:** Genia Lücking, Elrike Frenzel, Andrea Rütschle, Sandra Marxen, Timo D. Stark, Thomas Hofmann, Siegfried Scherer, Monika Ehling-Schulz

**Affiliations:** ^1^Department of Microbiology, Central Institute for Food and Nutrition Research (Zentralinstitut für Ernährungs- und Lebensmittelforschung), Technische Universität MünchenFreising, Germany; ^2^Functional Microbiology, Institute of Microbiology, Department of Pathobiology, University of Veterinary Medicine ViennaVienna, Austria; ^3^Chair of Food Chemistry and Molecular Sensory Science, Technische Universität MünchenFreising, Germany; ^4^Lehrstuhl für Mikrobielle Ökologie, Wissenschaftszentrum Weihenstephan, Technische Universität MünchenFreising, Germany

**Keywords:** *Bacillus cereus*, *ces* gene cluster, regulation, *cesH*, *cesP*, *cesC*, *cesD*, cereulide synthetase

## Abstract

The emetic toxin cereulide produced by *Bacillus cereus* is synthesized by the modular enzyme complex Ces that is encoded on a pXO1-like megaplasmid. To decipher the role of the genes adjacent to the structural genes *cesA/cesB*, coding for the non-ribosomal peptide synthetase (NRPS), gene inactivation- and overexpression mutants of the emetic strain F4810/72 were constructed and their impact on cereulide biosynthesis was assessed. The hydrolase CesH turned out to be a part of the complex regulatory network controlling cereulide synthesis on a transcriptional level, while the ABC transporter CesCD was found to be essential for post-translational control of cereulide synthesis. Using a gene inactivation approach, we show that the NRPS activating function of the phosphopantetheinyl transferase (PPtase) embedded in the *ces* locus was complemented by a chromosomally encoded Sfp-like PPtase, representing an interesting example for the functional interaction between a plasmid encoded NRPS and a chromosomally encoded activation enzyme. In summary, our results highlight the complexity of cereulide biosynthesis and reveal multiple levels of toxin formation control. *ces* operon internal genes were shown to play a pivotal role by acting at different levels of toxin production, thus complementing the action of the chromosomal key transcriptional regulators AbrB and CodY.

## Introduction

The cyclic dodecadepsipeptide cereulide, a heat-, acid-, and proteolytically stable toxin, is responsible for the emetic type of food borne illness caused by a specific subgroup of *Bacillus cereus*. Intoxication with cereulide, which is preformed during vegetative growth of *B. cereus* in foods, causes nausea and heavy vomiting around 0.5–6 h after consumption of contaminated food (Ehling-Schulz et al., 2004). These symptoms are presumably induced by the interaction of cereulide with 5-HT_3_ serotonin receptors leading to the stimulation of the afferent vagus nerve (Agata et al., 1995). Usually, these symptoms decline after 24 h, but more severe foodborne intoxications requiring hospitalization or even including fatalities are reported increasingly (Dierick et al., 2005; Naranjo et al., 2011; Messelhäusser et al., 2014; Tschiedel et al., 2015).

In agreement with its chemical structure [D-*O*-Leu-D-Ala-L-*O*-Val-L-Val]_3_, cereulide is produced enzymatically by the non-ribosomal cereulide peptide synthetase Ces (Ces-NRPS; Ehling-Schulz et al., 2005b). NRPSs are large multifunctional enzyme complexes consisting of repetitive modules which selectively incorporate amino acid, α-hydroxy acid or carboxylic acid monomers in the peptide product (Marahiel et al., 1997). The order of the modules usually corresponds directly to that of the monomers in the assembled peptide chain, although strict co-linearity is not always reinforced in nature (Wenzel and Müller, 2005; Lane and Moore, 2011). Very recently we could show that the enzymatic activity of the Ces-NRPS does not follow the canonical NRPS biosynthesis logic, but represents a novel mechanism of non-ribosomal peptide assembly, by using dipeptides rather than monomers as basic units (Marxen et al., 2015a,b).

**FIGURE 1:**
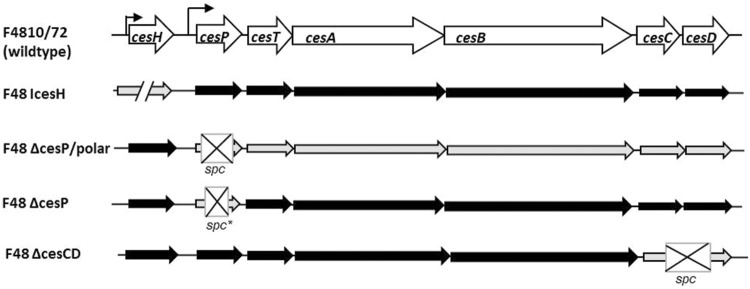
**Genetic organization of the cereulide biosynthetic gene cluster in F4810/72 wildtype and *ces* mutant strains used in this study.** The locus consists of seven open reading frames encoding a putative hydrolase/acetyltransferase (*cesH*), a phosphopantetheinyl transferase (*cesP*), a type II thioesterase (*cesT*), two non-ribosomal peptide synthetase modules (*cesA* and *cesB*) and a putative ABC transporter (*cesC* and *cesD*) (according to Ehling-Schulz et al., 2006; Dommel et al., 2010). Identified promoter regions are marked by bent arrows. Gene disruption is indicated by slashes, gene deletion, and introduction of a spectinomycin resistance cassette (spc) by crossed boxes. spc^∗^ represents a non-polar mutagenic spc cassette lacking promoter- and terminator sequences and followed by a ribosome binding site and a start codon.

The cereulide biosynthetic genes were found to be located on a pXO1-like megaplasmid, organized in a 24-kb cluster comprising the seven *ces* genes *cesH, cesP, cesT, cesA, cesB, cesC*, and *cesD* (Ehling-Schulz et al., 2006). The *cesPTABCD* genes represent an operon being transcribed as a single 23-kb polycistronic mRNA, whereas the adjacently located *cesH* is transcribed from its own promoter (Dommel et al., 2010; **Figure 1**). The structural NRPS genes *cesA* and *cesB* encode the modules that are responsible for the activation and incorporation of each two monomers: The CesA2 and CesB2 submodules install D-alanine and L-valine, respectively, whereas CesA1 and CesB1 bind α-keto acids of leucine and valine (α-ketoisocaproic acid and α-ketoisovaleric acid; Magarvey et al., 2006; Alonzo et al., 2015). A central condensation domain in the C-terminal part of CesA together with a type I thioesterase (TE) domain located in the C-terminal part of CesB is suggested to act as esterification and elongation center before the final dodecadepsipeptide cereulide is released from the TE domain by macrocyclization (Marxen et al., 2015b).

The adjacent to *cesAB* located gene *cesT* encodes a putative type II thioesterase (TEII), an enzyme which is often found in association with NRPSs. In contrast to the type I thioesterases, which are an integrated part of the NRPS catalyzing the product release, external TEIIs reactivate the catalytic NRPS domains by removing misprimed monomers (Schwarzer et al., 2002). The *cesP* gene, located upstream of *cesT*, encodes a putative 4′-phosphopanthetheinyl transferase (PPTase). Such enzymes are detected frequently in association with NRPS enzymes, since they are crucial for their activation. PPTases catalyze the transfer of a 4′-phosphopantheteine (Ppant) moiety of coenzyme A to a conserved serine residue of the PCP domain, thereby converting the *apo*-carrier protein to its active *holo-*form (Lambalot et al., 1996).

Besides these typical NRPS-associated genes, three additional open reading frames with so far unknown functions are present in the *ces* gene cluster: *cesH*, at the 5′ end, encodes a putative hydrolase or acyltransferase, whose role regarding cereulide synthesis is unclear. *cesC* and *cesD*, encoding a putative ABC transporter, are located at the 3′ terminus of the *ces* operon (Ehling-Schulz et al., 2006).

The aim of this work was to assess the role of the *ces* genes flanking the structural cereulide synthetase genes in order to gain further insights into the unusual biosynthetic pathway, which catalyzes the production of the highly potent depsipeptide toxin cereulide. An essential prerequisite for translational studies was the availability of a Ces-NRPS specific antibody, which was raised successfully against the CesB1 module. Targeted gene inactivation- and overexpression mutants of the emetic reference strain F4810/72 were constructed and characterized regarding the different steps of toxin synthesis. The influence of the accessory *ces* genes on the different cereulide process levels is demonstrated and resulting functional predictions are discussed.

## Materials and Methods

### Bacterial Strains, Plasmids and Growth Conditions

Details on bacterial strains and plasmids used in this study are provided in **Table 1** and Supplementary Table S1, respectively. Unless otherwise specified, all *B. cereus, Bacillus subtilis* and *Bacillus megaterium* were grown in LB broth (10 g/L tryptone, 5 g/L yeast extract, 10 g/L NaCl) or on LB agar plates at 30°C. For liquid cultures, 100 ml LB media was inoculated with approximately 10^3^ cfu/ml from a 14–16 h pre-culture and cultures were incubated in 500 ml baﬄed flasks with 150 rpm. *Escherichia coli* strains used for subcloning were cultured in LB at 37°C. Growth was monitored by optical density at 600 nm (OD_600_) using a GeneQuant pro spectrophotometer (Biochrom). Concentrations of antibiotics applied were 100 μg/ml for ampicillin, spectinomycin, and polymyxin B; 50 μg/ml for kanamycin; 5 μg/ml for erythromycin; 5 μg/ml for chloramphenicol; and 10 μg/ml for tetracycline.

**Table 1:** Bacterial strains used in this study.

Strain	Relevant characteristics	Reference
***E. coli***		
TOP10	General cloning host	Invitrogen
INV110	Methylase-deficient general cloning host	Invitrogen
JM83/pRK24	Donor strain for conjugation; Tra+, Mob+, Amp^r^, Tc^r^	Trieu-Cuot et al., 1987
***B. subtilis***		
168		Spizizen, 1958
***B. cereus***		
ATCC 14579	Type strain (non-emetic)	Ivanova et al., 2003
ATCC 10987	Emetic-like strain	Rasko et al., 2004
A529	Emetic food-borne outbreak isolate	Stark et al., 2013
F4810/72	Emetic reference strain (also AH 187)	Ehling-Schulz et al., 2005a
F48ΔcesP/polar	F4810/72 Δ*cesP::spc*; Spc^r^	This study
F48ΔcesP	F4810/72 Δ*cesP::spcH+2* (non-polar Spc resistance cassette); Spc^r^	This study
F48ΔcesCD	F4810/72 Δ*cesCD::spc;* Spc^r^	This study
F48ΔcesCD/com_cesCD	F48ΔcesCD complemented with pAD/Pro-ces/cesCD; Spc^r^, Cm^r^	This study
F48IcesH	F4810/72 with disrupted *cesH (cesH::pMAD)*; Ery^r^	This study
F48Ippt	F4810/72 with disrupted *ppt (ppt::pMAD);* Ery^r^	This study
F48ΔcesP/Ippt	F48ΔcesP with disrupted *ppt*; Spc^r^, Ery^r^	This study
F48ΔcesP/Ippt/com_cesP	F48ΔcesP/Ippt complemented with pAD/Pro-ces/cesP; Spc^r^ Ery^r^, Cm^r^	This study
F48ΔcesP/Ippt/com_ppt	F48ΔcesP/Ippt complemented with pMM/ppt; Tc^r^	This study
F48pMM/cesH	F4810/12 containing pMM/cesH for CesH overexpression; Tc^r^	This study
***B. megaterium***		
WH320	Protein overexpression strain lacking alkaline proteases	MoBiTec
WH320pWHCesB1His	WH320 containing pWHCesB1His for CesB overexpression; Amp^r^, Tc^r^	This study

### Sequence Analysis

The genome information of the emetic reference strain F4810/72 (AH187), retrieved from the NCBI website (GenBank accession no. CP001177 and CP001179), was used for sequence analysis of the *ces* locus and homology searches. Amino acid sequences were retrieved from the NCBI database and protein homology search was performed using Blastp available at NCBI^1^. Multiple sequence alignments were carried out with Clustal W using default parameters (Thompson et al., 1994). Maximum-likelihood trees were constructed with MEGA 5 (Tamura et al., 2011) using the Jones–Thornton–Taylor (JTT) model. All positions containing gaps were eliminated. Numbers at nodes (≥50%) represent bootstrap support of 500 resamplings. Annotation of characteristic protein domains and membrane spanning motifs was achieved with the SMART database (Letunic et al., 2012) and the programs TMHMM^2^ and TMpred^3^.

### Construction of *B. cereus* ΔcesP and ΔcesCD Deletion Mutants

Deletion and complementation mutants were constructed as described previously (Lücking et al., 2009). In brief, flanking regions of *cesP* and *cesCD* were amplified by PCR using the primer pairs listed in Supplementary Table S2. Digested PCR products, together with an excised spectinomycin resistance cassette (spc) from pUC1318spc (Murphy, 1985), were ligated into the TOPO pCR 2.1 vector (Invitrogen). For *cesP*, an additional construct with a non-polar mutagenic spectinomycin cassette from pSPCH+2 (Mesnage et al., 2000) was produced to avoid polar effects of the spectinomycin cassette on the *ces* operon genes located downstream of *cesP*. Constructs were excised from TOPO and inserted into the conjugative suicide vector pAT113, giving rise to pAT113ΔcesP/spc, pAT113ΔcesCD/spc, and pAT113ΔcesP/spcH+2 (see Supplementary Table S1 for details). Plasmids were then transformed into *E. coli* JM83/pRK24 by heat shock and the resulting strains were used for transconjugal transfer into *B. cereus* F4810/72 using a mating procedure previously described (Pezard et al., 1991). For complementation of F48ΔcesCD and F48ΔcesP/Ippt, the genes *cesCD* and *cesP* were amplified using the primers cesCD_F_Xba/cesCD_R_Pae and cesP_Pro_F2/cesP_Pro_R (Supplementary Table S2), respectively, and cloned into the shuttle vector pAD123 together with the ∼500 bp *ces* promoter region. For complementation of F48ΔcesP/Ippt with *ppt* (NCBI accession no. ACJ79141), the respective gene was amplified with ppt_chr_F and ppt_chr_R and ligated into the shuttle vector pMM1522, containing a xylose-inducible promoter. Constructs were propagated in the non-methylating *E. coli* strain INV110 cells and then introduced into the according *B. cereus* mutant by electroporation, giving rise to F48ΔcesCD/com_cesCD, F48ΔcesP/Ippt/com_cesP, and F48ΔcesP/Ippt/com_ppt.

### Construction of *B. cereus cesH* and *ppt* Insertion- and Overexpression Mutants

Gene inactivation by pMAD integration was performed as described previously (Dommel et al., 2011). In brief, internal fragments (∼300 bp) of *cesH* and *ppt* (NCBI accession no. ACJ79141) were amplified by PCR using primers listed in Table S2 and ligated into the thermosensitive shuttle vector pMAD. Constructs were introduced into *B. cereus* F4810/72 or F48ΔcesP by electroporation and transformants were obtained after 2 days at 30°C on LB plates with erythromycin and X-Gal (20 μg/ml). Plasmid integration was enforced by re-cultivation of a blue colony in LB medium overnight at 42°C for several times. For further experiments, the resulting strains F48IcesH, F48Ippt, and F48ΔcesP/Ippt were cultured at high temperatures (37 or 42°C), which is essential to avoid the loss of the integrated plasmid.

For *cesH* overexpression, the promoterless *cesH* gene was amplified using the respective primers listed in Supplementary Table S2. The digested fragment was ligated into pMM1522, harboring a xylose-inducible promoter, and then introduced in *B. cereus* F4810/72 by electroporation. To induce gene overexpression, 0.1 % D-xylose (v/v) was added to the culture medium.

### Production of a Monoclonal anti-CesB Antibody

For generation of a monoclonal antibody targeting the cereulide NRPS, the DNA fragment (3999 bp) encoding the first CesB submodule (CesB1: consisting of an adenylation-, ketoreductase-, and peptidyl carrier protein domain) including the native *cesB* ribosome binding site was amplified with Pfu polymerase (Promega) from genomic DNA of *B. cereus* F4810/72 using the primer pair cesB135Nco_for/cesB135Xho_rev (Supplementary Table S2). Cloning *via* the XhoI/NcoI sites into the pET28b(+) vector resulted in a transcriptional C-terminal His_6_ tag fusion. CesB1-His_6_ was amplified from pET28-*cesB1* by PCR with cesBpETSpe_for2 and cesBpETSph_rev2 and cloned into the SpeI and SphI restriction sites of pWH1520, giving rise to the xylose-inducible overexpression plasmid pWHCesB1His. This construct was transferred into *B. megaterium* WH320 protoplasts by PEG-fusion according to the manufacturer’s protocol (MoBiTec, Göttingen). The CesB1His_6_ protein was overexpressed in recovered *B. megaterium* cells and purified using Ni-NTA affinity columns (Qiagen) according to the manufacturer’s instruction. The monoclonal antibody (mAB) CesB5-6 was raised by BioGenes (Berlin) using the purified CesB1His_6_ protein, NMRI mice (obtained from Janvier, France), and the myeloma cell line SP2/0-Ag14 (obtained from DSMZ, Germany). Purified CesB1 mAB with a concentration of 1.68 μg ml^-1^ in PBS solution was tested for its specificity by enzyme-linked immunosorbent assays (ELISA; data not shown) and by immunoblotting.

### Reverse Transcription-qPCR

To analyze *cesA* transcript levels, 1 ml culture samples of *B. cereus* strains were harvested at OD_600_ 8 by centrifugation (10000 *g*, 4°C, 2 min). Total RNA isolation, cDNA synthesis and RT-qPCR were carried out as described previously (Dommel et al., 2011). Transcript levels of the 16S *rrn* gene served as the reference control for data normalization and relative gene expression ratios were calculated with the REST software (Pfaﬄ et al., 2002).

### Western- and Slot Blot Analysis of CesB

Twenty milliliter of *B. cereus* cultures, grown to different optical densities, were centrifuged (9500 rpm, 6 min, Sigma 3–18K with angle rotor 19776) and cell pellets resuspended in 2 ml lysis buffer [50 mM Tris-HCl pH 7.6, 2 mM EDTA pH 7.5, 1 mM Pefabloc (Merck)]. Cells were disrupted by two passages through a French Pressure cell press (1000 psi) and the soluble protein fraction was collected by centrifugation of the lysates (13000 rpm, 2x 30 min, 4°C). Protein concentration was determined according to Bradford using the Roti-Quant solution (Carl Roth GmbH). For Western blot analysis, 30 μg of total protein was separated by SDS-PAGE on 8% gels and the proteins were immobilized onto a PVDF membrane (Millipore) by semi-dry blotting for 2 h at 120 mA. A 1:2000 dilution of the monoclonal anti-CesB antibody was used to detect the CesB1 module. The second incubation was done with a 1:20000-dilution of HRP-conjugated goat anti-mouse IgG (Dianova) and blots were developed with the SuperSignal West Pico Chemiluminescent Substrate (Thermo Scientific). For slot blot analysis, 25 μg of total protein was blotted directly onto a PVDF membrane by vacuum filtration. Again, the 1:2000 dilution of the monoclonal anti-CesB antibody was used to detect the CesB module of the cereulide synthetase. In addition, blots of the same samples were performed with a 1:5000 dilution of a polyclonal rabbit anti-AtpB serum (Agrisera) detecting the beta subunit of the ATP synthase. As second antibodies served an alkaline phosphatase-conjugated goat anti-mouse IgG (1: 10000 dilution, Dianova) and an alkaline phosphatase-conjugated goat anti-rabbit IgG (1:3000, Dianova), respectively. Chromogenic detection of alkaline phosphatase activity was accomplished with BICP-p-toluidine salt- and NBT solutions (Carl Roth GmbH) according to the manufacturer’s instructions.

### Sample Preparation and Cereulide Quantification by Means of UPLC-MS/MS

The biosynthetic production of cereulide in LB broth supplemented with 0.2% glucose and of ^13^C-labeled cereulide in MOD medium supplemented with ^13^C_1_-L-valine was carried out as described previously (Bauer et al., 2010). Extraction of cereulide from autoclaved cells of different *B. cereus* strains grown in 100 ml LB broth was carried out as described (Stark et al., 2013), using 1 ng of ^13^C_6_-cereulide as internal standard. Cereulide quantification via stable isotope dilution analysis (SIDA) was performed using a Xevo TQ-S Acquity i-class UPLC-MS/MS system and an UPLC BEH C18 column (2.1 mm × 50 mm, 1.7 μm; Waters, UK and USA). One microliter aliquots of ethanolic extracts (of *B. cereus* samples) were injected directly into the UPLC/MS-MS system. Operated with a flow rate of 1.0 ml/min at a temperature of 50°C, the following gradient was used for chromatography: starting with a mixture (90/10, v/v) of methanol and 10 mM ammonium formate (0.1% formic acid), the methanol content was increased to 100% within 0.5 min, kept constant for 0.3 min and decreased within 0.1 min to 90%. Measurements were performed using electrospray with positive ionization and the quantitative calibration mode consisting of the following ion source parameters: capillary voltage +3.5 kV, sampling cone 30 V, source offset 30 V, source temperature 150°C, desolvation temperature 600°C, cone gas 150 L/h, desolvation gas 1000 L/h, collision gas flow 0.15 ml/min and nebuliser gas flow 6.5 bar. Calibration of the TQ-S in the range from *m/z* 40-1963 was performed using a solution of phosphoric acid (0.1% in acetonitrile). The UPLC and Xevo TQ-S systems were operated with MassLnyx^TM^ 4.1 SCN 813 software, data processing and analysis were performed using TargetLynx. By means of the multiple reaction monitoring (MRM) mode, the ammonium adducts of cereulide (qualifier: *m/z* 1170.7→172.2; 1170.7→314.2; quantifier: *m/z* 1170.7→357.2) and ^13^C_6_-cereulide (*m/z* 1176.7→173.2; 1176.7→316.3; 1176.7→358.3) were analyzed using the mass transitions (given in brackets) monitored for a duration of 25 ms. ESI^+^ mass and product ion spectra were acquired with direct flow infusion using IntelliStart. The MS/MS parameters were tuned for each individual compound, detecting the fragmentation of the [M+NH_4_]^+^ molecular ions into specific product ions after collision with argon. For quantitation, 10 ethanolic standard solutions of the analyte cereulide (0.05–10.0 ng/ml) and the internal standard ^13^C_6_-cereulide (1 ng/ml) were mixed and analyzed in triplicates by means of UPLC–MS/MS using the MRM mode. Calibration curve was prepared by plotting peak area ratios of analyte to internal standard against concentration ratios of each analyte to the internal standard using linear regression.

### Cytotoxicity Test

Aliquots of *B. cereus* cultures were taken after 24 h of incubation and autoclaved (15 min at 121°C) to lyse cells and denature heat-labile toxins. Cereulide amounts of these samples were determined using the HEp-2 cell based cell culture assay as previously described (Lücking et al., 2009). Cereulide titres of the mutant strains were normalized to the value of the parental strain *B. cereus* F4810/72 (grown for 24 h at 30 or 37°C, respectively), which was defined as being 100%.

## Results

### Comparative Sequence Analysis of the Cereulide Synthetase Flanking Genes

Based on the genome information (NCBI accession no. CP001177 and CP001179) available for the emetic reference strain F4810/72 (AH187), a BLAST database search and extensive sequence analysis was carried out. This *in silico* approach reconfirmed the *ces* gene cluster boundaries suggested previously (Ehling-Schulz et al., 2006; Dommel et al., 2010). The *ces* gene locus comprises seven genes (*cesHPTABCD*), which are present in all emetic *B. cereus* and emetic *Bacillus weihenstephanensis* sequenced so far (data not shown). *cesH* represents the 5′ prime end of the *ces* locus while *cesD*, which is followed by a strong terminator sequence, marks the 3′ prime end.

**FIGURE 2:**
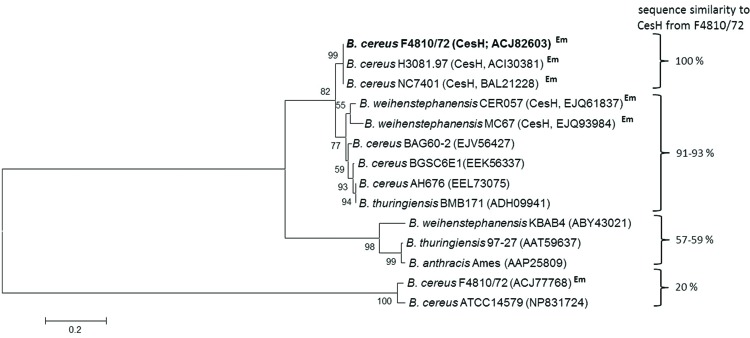
**Maximum-likelihood protein similarity tree based on amino acid sequences of CesH from *Bacillus cereus* F4810/72 (AH187) and selected homologs of other *B. cereus* group members.** NCBI accession numbers of proteins are given in brackets. Em indicates an emetic strain containing the *ces* gene cluster.

CesH is a putative 31 kDa hydrolase belonging to the α/β-hydrolase fold superfamily of proteins. Besides the plasmid-borne *cesH*, F4810/72 carries 13 chromosomal genes annotated to code for members of this hydrolase subfamily, with none of them showing notable sequence similarity toward CesH. Comparison of the CesH with amino acid sequences retrieved from annotated genomes of the *B. cereus* group members revealed a large number of orthologs showing significant similarity. However, proteins with 100% identity (amino acids) were only found in emetic *B. cereus* strains possessing the *ces* gene cluster. The CesH sequences retrieved from the *ces* gene cluster in emetic *B. weihenstephanensis* strains showed a homology of 91–93% (**Figure 2**).

**FIGURE 3:**
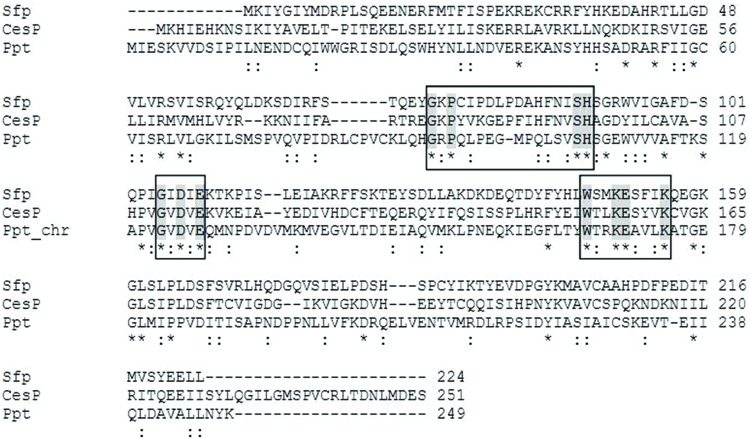
**Amino acid sequence alignment of the PPTases CesP (ACJ82723), Ppt (ACJ79141) of *B. cereus* F4810/72, and Sfp (P39135) of *B. subtilis*.** Three conserved motifs, which are typical for members of the “Sfp-like” PPTase subfamily, are shown in boxes. Identical residues are highlighted with asterisk, similar residues with colon.

*cesP* codes for a putative 4′-phosphopantetheinyl transferase (PPtase) belonging to the Sfp-like subgroup of the PPTase superfamily. *In silico* analysis revealed the presence of an additional gene encoding a 4′-PPtase (ACJ79141; herein after referred to as *ppt*) in the chromosome of *B. cereus* F4810/72. A homolog of this protein with similarity above 80% is present in almost all *B. cereus* group members, while CesP homologs can only be found in emetic *B. cereus* and emetic *B. weihenstephanensis* strains. An alignment of the amino acid sequences of CesP and Ppt resulted in a rather low degree of homology (27% identity at 56% coverage), nevertheless, according to their size and three conserved motifs, both PPTases can be classified as typical members of the “Sfp-like” family (Copp and Neilan, 2006), showing 32% (CesP) and 26% (Ppt) identity to Sfp (P39135), the prototype PPTase of *B. subtilis*, respectively (**Figure 3**).

**FIGURE 4:**
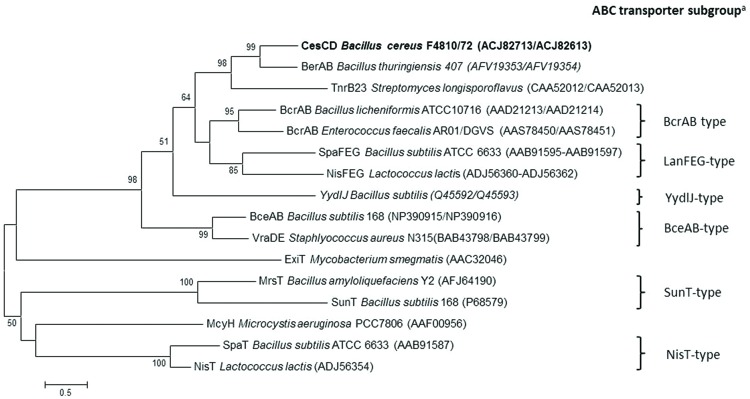
**Protein similarity tree of the amino acid sequences of CesCD and previously characterized ABC transporters.** ^a^Classification of ABC transporters of antimicrobial peptides according to Gebhard (Gebhard, 2012).

The two open reading frames *cesC* and *cesD* at the 3′ terminus of the *ces* gene locus encode a putative ABC transporter. Protein domain annotation using the SMART database (Letunic et al., 2012) revealed the presence of characteristic motifs involved in ATP- binding and hydrolysis for CesC. The amino acid sequence of CesC was found to be homologous to several known ATP-binding proteins, e.g., BerA of *B. thuringiensis* required for β-exotoxin I production (Espinasse et al., 2002), or TnrB2 of *Streptomyces longisporoflavus* and BcrA of *B. licheniformis*, which have been shown to confer resistance to tetronasin and bacitracin, respectively (Linton et al., 1994; Podlesek et al., 1995; Supplementary Figure S1). For CesD, five hydrophobic transmembrane domains were predicted, which are typical for membrane-spanning proteins. CesD showed homology (33–34% identity) only toward the putative permease BerB of *B. thuringiensis* and its orthologs present in many *B. cereus* group members. Sequence alignment of CesCD with known ABC transporters of antimicrobial peptides confirmed that CesCD most closely resembles members of the BcrAB subgroup, which generally consist of one ATPase and one permease with six transmembrane helices (Gebhard, 2012; **Figure 4**).

### Construction of Mutants and Determination of their Cereulide Production Capacities

To investigate the potential role of *cesH, cesP, cesC*, and *cesD* on cereulide production, various deletion-, insertion-, and overexpression mutants of *B. cereus* F4810/72 were constructed as described in the materials and methods section. An overview of mutants generated is provided in **Figure 1** and the major genetic characteristics of mutant strains are summarized in **Table 1**, respectively. After growth in LB medium for 24 h at 30 or 37°C (insertion mutants), culture samples were taken and cells disrupted by autoclaving in order to obtain total cereulide amounts including extra- and intracellularly accumulated toxin. Cereulide concentrations of ethanolic extracts were measured by means of SIDA-UPLC-MS/MS. Furthermore, cytotoxicity was determined by the HEp-2 cell culture assay.

The cereulide production of the *cesH* inactivation mutant (F48IcesH) turned out to be almost twice as high compared to the wildtype strain. Interestingly, when *cesH* was overexpressed in the wildtype strain (F48pMM/cesH) with a xylose-inducible shuttle vector, almost no cereulide was detectable, while in the absence of the inducer xylose, cereulide production was comparable to the wildtype (**Table 2**). These results demonstrate that CesH inhibits cereulide synthesis.

**Table 2:** Cereulide production of wildtype emetic *B. cereus* and *ces* gene mutants determined by SIDA-UPLC-MS/MS and HEp-2 bioassay.

Strain	Cereulide concentration in μg/ml	Cytotoxicity in %^c^
Wildtype (F4810/72)^a^	4.28 ± 1.06	100
Wildtype (F4810/72)^b^	3.67 ± 0.70	100
F48IcesH^b^	5.76 ± 0.99	106.6 ± 23.2
F48pMM/cesH (+0.1% Xyl)^a^	0.04 ± 0.01	1.3 ± 0.6
F48pMM/cesH (w/o Xyl)^a^	3.47 ± 1.59	97.6 ± 37.9
F48ΔcesP/polar^a^	0.0 ± 0.0	0.0 ± 0.0
F48ΔcesP^a^	3.54 ± 1.07	83.2 ± 23.8
F48Ippt^b^	3.19 ± 1.62	75.4 ± 26.4
F48ΔcesP/Ippt^b^	0.0 ± 0.0	0.9 ± 1.9
F48ΔcesP/Ippt/com_cesP^b^	2.62 ± 1.51	99.9 ± 44.6
F48ΔcesP/Ippt/com_ppt^b^	0.49 ± 0.02	32.6 ± 15.3
F48ΔcesCD^a^	0.0 ± 0.0	0.0 ± 0.0
F48ΔcesCD/com_cesCD^a^	0.87 ± 0.20	43.7 ± 2.4

*^a^Strain grown at 30°C.*

*^b^Strain grown at 37°C.*

*^c^Values normalized to cytotoxicity of wildtype at corresponding temperature.*

For the analysis of *cesP*, two knockout strains were generated, since *cesP* is the first gene of the *ces* operon and disruption of this gene may affect the *ces* genes located downstream. Thus, in one strain a non-polar spectinomycin resistance cassette (spc) lacking promoter and terminator sequences (Mesnage et al., 2000) was used for mutagenesis (F48ΔcesP), while the other harbors a normal spc potentially leading to polar effects on downstream located genes (F48ΔcesP/polar; see also **Figure 1**). Indeed, SIDA-UPLC-MS/MS of the latter revealed no cereulide production and no toxicity could be detected toward HEp-2 cells. In contrast, F48ΔcesP produced toxin amounts similar to that in the wildtype, demonstrating *cesP*-independent cereulide synthesis (**Table 2**). Since *in silico* analysis exposed the presence of a second sfp-like PPTase in the chromosome of *B. cereus* F4810/72 (*ppt*), a respective gene inactivation mutant was constructed in the wildtype (F48Ippt) to test its possible involvement in toxin formation. UPLC-MS/MS showed that this mutant produces cereulide levels comparable to the wild type. However, when *ppt* was disrupted in F48ΔcesP, leading to a double knock out mutant in *cesP* and *ppt* (F48ΔcesP/Ippt), no cereulide or cytotoxicity was detected (**Table 2**). This effect was reversible by complementation of the mutant with *cesP* and – to a lesser extend – with *ppt*, indicating a redundant role of CesP and Ppt in cereulide production.

For the *cesCD* deletion mutant (F48ΔcesCD) neither cereulide by means of MS nor cytotoxicity in the HEp-2 bioassay could be determined. Both parameters could be restored to some extend by *in trans* complementation of F48ΔcesCD with a shuttle vector containing *cesC* and *cesD* under the control of the *ces* locus promoter (**Table 2**). These results demonstrate that the putative ABC transporter genes *cesC* and *cesD* are essential for cereulide formation in *B. cereus* F4810/72.

### Generation of a Monoclonal Antibody Targeting the Cereulide NRPS

Next, we were interested to further dissect the level of regulation leading to the huge differences in cereulide quantities found in the *ces* mutants. To study the impact of the *ces* mutations on the expression of the Ces-NRPS, a specific antibody against the cereulide synthetase was generated. The CesB1 submodule of the cereulide synthetase, which was shown to present a ketoacid-binding module unique among *Bacillus* sp. NRPS (Magarvey et al., 2006), served as antigen for the generation of monoclonal antibodies (mAB) as outlined in the materials and methods section. The specificity of the resulting monoclonal anti-CesB1 antibody was assessed using two emetic-, two non-emetic *B. cereus* strains and one *B. subtilis* strain (possessing the surfactin NRPS genes) by ELISA (data not shown) and immunoblotting. Western blot analysis revealed the specificity of the antibody toward the two cereulide-positive strains *B. cereus* F4810/72 and A529, while no signal was detected for the cereulide-negative strains ATCC 10987 and ATCC 14579 or for *B. subtilis* 168 (Supplementary Figure S2). The CesB1 mAB reacted specifically with a large protein band migrating way above the range of the protein ladder used, which is consistent with the predicted molecular mass of CesB1 at 304 kDa. The appearance of an additional weak band at 170 kDa indicates fragmentation of the large CesB1 complex, which was repeatedly observed after denaturing PAGE.

### Transcriptional and Translational Analysis of *ces* Mutant Strains

**FIGURE 5:**
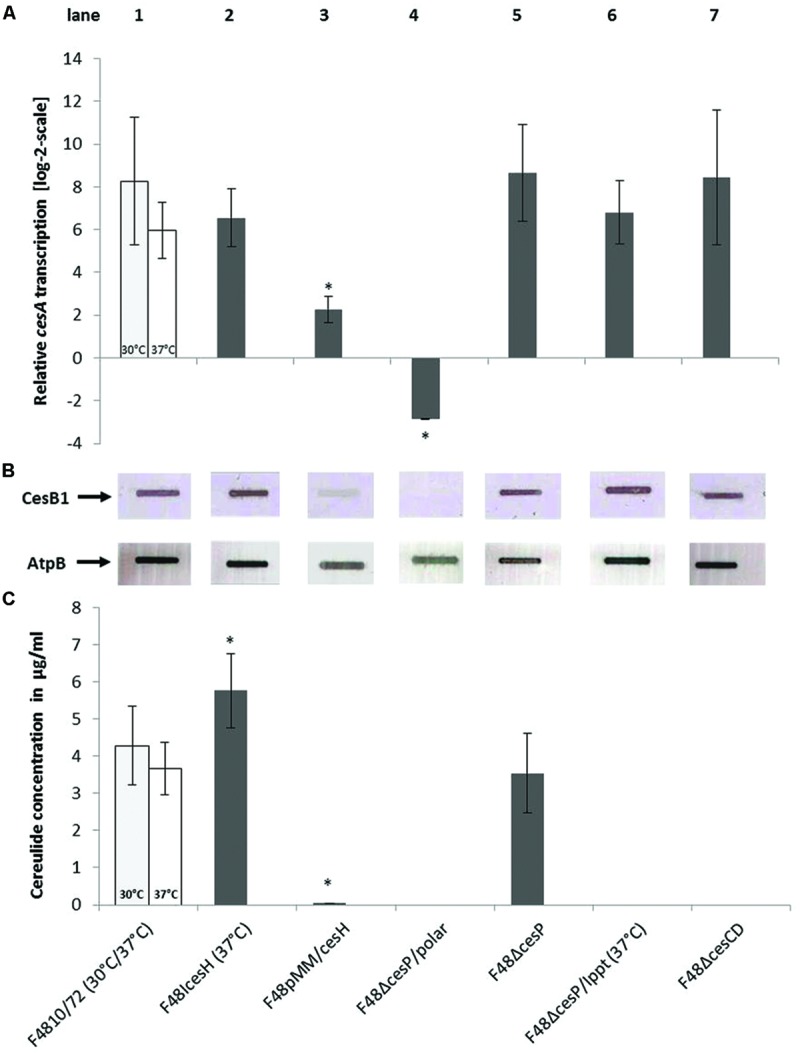
**Comparison of *ces* gene transcription, cereulide synthetase expression, and cereulide production of *B. cereus* F4810/72 wildtype (WT; 1) and the mutant strains F48ΔcesP (5), F48ΔcesP/Ippt (6), F48ΔcesP/polar (4), F48ΔcesCD (7), F48IcesH (2), and F48pMM/cesH (3). (A)** Relative *cesA* transcription of *B. cereus* strains grown in LB medium at 30 or 37°C (insertion mutants) and harvested at exponential growth phase (OD_600_ = 8) for RNA isolation. The asterisks denote statistically significant (*P* < 0.05) differences in *cesA* mRNA levels between wildtype and mutant strains grown under the same conditions. **(B)** Slot blot analysis of CesB and AtpB expression using 25 μg of total protein per slot from *B. cereus* strains harvested at OD_600_ = 8. The CesB1 module of the cereulide synthetase was detected with the CesB1 mAB and AtpB (beta subunit of ATP synthase) with a polyclonal anti-AtpB antibody, serving as a protein loading control. **(C)** Cereulide production of *B. cereus* strains determined by UPLC-MS/MS-SIDA. Strains were incubated in LB medium for 24 h (stationary growth phase) before cereulide was extracted for quantification. Statistically significant (*P* < 0.05) differences in cereulide concentration between wildtype and mutant strains are marked by asterisks.

Quantitative RT-PCR detecting *cesA* mRNA levels as well as immunoblotting using the CesB mAB were carried out to investigate, whether the striking differences of cereulide synthesis of the *B. cereus* F4810/72 wildtype and the *ces* mutants (**Table 2** and **Figure 5C**) were caused by variations in NRPS gene transcription or by translational regulation of the synthetase complex. **Figure 5** combines the results of *ces* transcription-, translation-, and cereulide production analyses for the wildtype and seven mutant strains. For the cereulide-negative mutant F48ΔcesP/polar, no *cesA* transcripts or CesB expression was detectable (**Figures 5A,B**, lane 4), confirming the assumption that insertion of spc in *cesP* led to a transcriptional stop of the downstream located *ces* genes in the operon. In contrast, transcript levels and CesB protein signals for the other two cereulide-deficient mutants, F48ΔcesP/Ippt and F48ΔcesCD, resembled those of the parental strain (**Figures 5A,B**, lanes 6 and 7). These data suggest that *cesP, ppt, cesCD* and their respective gene products do not interfere with *ces* transcription or translation of the cereulide synthetase complex, but seem to play a role in the post-translational formation of active cereulide. Transcript- and CesB protein levels of the *cesH* insertion mutant (F48IcesH), which revealed higher toxin levels than the wildtype in the MS assay (**Figure 5C**, lane 2), were comparable to those of the wildtype. However, for the *cesH* overexpression mutant (F48pMM/cesH), *cesA* transcription was down-regulated significantly and only a very weak CesB band was detected by slot blot analysis (**Figures 5A,B**, lane 3). Therefore, the toxin deficiency of the latter strain may be due to *cesH* and its gene product, acting as a possible transcriptional repressor of the cereulide synthetase genes.

## Discussion

Hitherto, research on the cereulide biosynthetic *ces* operon focused on the molecular and biochemical characterization of the non-ribosomal cereulide peptide synthetase encoded by *cesA* and *cesB* (Ehling-Schulz et al., 2005b; Magarvey et al., 2006; Alonzo et al., 2015; Marxen et al., 2015b). However, little is known about the adjacent genes *cesH, cesP, cesC*, and *cesD*, which code for a putative hydrolase, a PPtase and a putative transport system of the ABC-type, respectively (Ehling-Schulz et al., 2006). So far it is unclear, why different strains of the emetic *B. cereus* group display highly variable toxicity (Carlin et al., 2006; Stark et al., 2013). Thus, it is important to dissect the role of *ces* locus genes and their gene products on cereulide formation. Since it was recently shown that the Ces-NRPS represents a novel mechanism for non-ribosomal depsipeptide assembly (Marxen et al., 2015b), information on the functional architecture of the *ces* gene locus would also improve our general understanding of the complex biochemical pathways involved in the production of natural, non-ribosomally synthesized peptides.

### CesH, a Putative Repressor Involved in Timing of *ces* Gene Transcription

The first gene in the 5′ prime proximity of the *ces* cluster, *cesH*, codes for a putative hydrolase of the α/β-hydrolase fold superfamily. According to the ESTER classification database, *cesH* belongs to the 6_AlphaBeta_hydrolase subgroup, which to date consists of over 3800 members using various substrates (Hotelier et al., 2004). Although *cesH* is transcribed from its own promoter – while the remaining *ces* genes are co-transcribed – it is considered to be an integral part of the *ces* gene locus, since *in silico* analysis revealed a copy in all emetic *B. cereus* strains as well as in cereulide-producing *B. weihenstephanensis* strains. In general, the presence of hydrolase genes in NRPS gene clusters seems to be rare and their function is unknown. We found one gene coding for a CesH – homolog (presenting 59% identity) in close proximity of a 30 kb polyketide-NRPS locus of the non-emetic *B. weihenstephanensis* KBAB4. Surprisingly, our data revealed an inhibitory effect of CesH on cereulide formation, as CesH overexpression led to a non-toxic phenotype, while the *cesH* insertion mutant produced more cereulide than the wildtype (**Table 2**). Transcriptional analysis of the CesH overexpression mutant resulted in strongly down-regulated *cesA* mRNA levels, which is in line with the very weak CesB slot blot signal obtained for this mutant. These data indicate that CesH is an additional regulatory member of the cereulide pathway by acting directly or indirectly as a transcriptional repressor of the *ces* gene operon. Our previous work demonstrated that transcription of the polycistronic *cesPTABCD* genes is co-regulated in a complex manner by several key transcription factors of the chromosome (Ehling-Schulz et al., 2015), e.g., AbrB and CodY, leading to a tightly regulated transcription peak in late exponential phase (Lücking et al., 2009; Frenzel et al., 2012). While AbrB and CodY are responsible for the onset and strong increase of *cesPTABCD* transcription, CesH may function as a closing signal shutting down mRNA synthesis. Since a direct action of a hydrolase as a transcriptional regulator seems to be unlikely, another possibility may be that CesH acts indirectly by degrading metabolites or quorum sensing signaling molecules that influence *ces* gene transcription in later growth phases. This hypothesis is in line with transcriptional kinetic studies of *cesH* in *B. cereus* F4810/72, demonstrating highest *cesH* expression in stationary growth phase (Supplementary Figure S3B), while the other *ces* operon genes are known to be transcribed earlier in the growth cycle (Dommel et al., 2011). Further experiments including activity tests with purified CesH protein, which are clearly beyond the scope of the current study, are necessary to elucidate the regulatory role of *cesH* in cereulide production in detail.

### Plasmid Encoded CesP and Chromosomally Encoded Ppt are Two Sfp Type- PPTases with Functionally Redundant Enzymatic Activities

In contrast to the frequency of proteins from the α/β-hydrolase fold superfamily, only very few members of the PPtase superfamily are present in *Bacillus* species. By post-translational modification and thereby activation of acyl-, aryl-, or peptidyl- carrier proteins, PPTases are essential enzymes for the synthesis of fatty acids, polyketides and non-ribosomal peptides (Walsh et al., 1997). Based on size, conserved sequence motifs and substrate selectivity, bacterial PPTases can be classified into two major groups, the Sfp-like PPTases and the acyl carrier protein synthases (AcpS; Finking and Marahiel, 2004). While PPTases of the Sfp-type are mostly found in association with NRPS, the enzymes of the AcpS type are linked to the activation of fatty acid and polyketide synthesis. Genome data analysis of *B. cereus* F4810/72 revealed the presence of two chromosomally encoded PPTases: an AcpS-type PPtase (ACJ81272) and a Sfp-like PPTase (ACJ79141, here named Ppt). The latter is encoded in the vicinity of the *dhb* operon, which has been shown to be responsible for the non-ribosomal synthesis of the siderophore bacillibactin in *B. subtilis* (May et al., 2001). The plasmid-encoded *cesP* gene codes for an additional Sfp-like PPTase and presents an integral part of the *ces* gene cluster, being co-transcribed with the NRPS genes (Dommel et al., 2010). Surprisingly, CesP was not essential for cereulide synthesis, as deletion of *cesP* did not significantly alter cereulide production or toxicity of the strain. Only an additionally disrupted *ppt* led to a toxin-negative phenotype, indicating that Ppt can function as a redundant CesP-PPTase in cereulide biosynthesis. Likewise, CesP seems to be able to functionally complement Ppt, as siderophore production (measured by a colorimetric assay) was detectable in low amounts in mutant strains concerning solely *ppt* or *cesP*, but not in the double knockout strain (data not shown). Transcriptional analysis of *ppt* in *B. cereus* F4810/72 revealed a weak, but constitutive expression throughout growth with a slight peak in stationary phase (Supplementary Figure S3A), similar to *sfp* transcription in *B. subtilis*, which was shown to be weak according to low promoter activity (Nakano et al., 1992). In contrast, transcription of *cesP*, which is linked to the *ces* operon, is strongly growth phase dependent, peaking highly in the late logarithmic phase (Dommel et al., 2011). Thus, we propose that the two PPtases encoded by *cesP* and *ppt* present functionally redundant enzymes with temporal different expression profiles in the growth cycle of *B. cereus* F4810/72.

### ABC Transporter CesCD is Essential for Post-translational Cereulide Formation

Genes coding for transport systems of the ABC-type are frequently found part of, or in close proximity to, peptide synthetase operons of NRPS products, such as siderophores, antibiotics or lipopeptides. For example the production of lichenysin A of *B. licheniformis*, tyrocidine of *B. brevis*, syringomycin *of Pseudomonas syringae* or pyoverdine *of P. aeruginosa* are linked to different ABC transporters, which are thought to be involved in product secretion or self-resistance (Quigley et al., 1993; McMorran et al., 1996; Mootz and Marahiel, 1997; Yakimov et al., 1998). Gene disruption of the transporter genes *cesC* and *cesD* of *B. cereus* F4810/72 resulted in a complete cereulide deficient phenotype (**Table 2**), which could be restored partly by *in trans* complementation with *cesCD*, indicating that the putative ABC transporter is essential for toxin production. Sequence analysis of CesCD revealed homology to members of the BcrAB- or DRI- (drug resistance and immunity) subfamily of ABC systems (Dassa and Bouige, 2001; Davidson et al., 2008; Gebhard, 2012) with highest similarity toward the BerAB transporter of *B. thuringiensis*, which was shown to be essential for β-Exotoxin I production and suggested to cause toxin immunity by eﬄux of the molecule; but no experimental proof has been presented so far (Espinasse et al., 2002). Thus, it is tempting to speculate that CesCD is involved in the transport of the emetic toxin, maybe mediating its eﬄux and thereby conferring resistance. However, if CesCD were only responsible for cereulide export, an intracellular accumulation of toxin would be expected by inactivation of the transporter. Since no accumulation, but complete abolition of cereulide was observed in the CesCD knockout mutant, we propose that CesCD, besides having a potential transport function, plays a more direct role in the cereulide biosynthesis pathway without influencing *ces* gene transcription or NRPS translation. For the lantibiotics nisin and subtilin it was shown that the multimeric synthetase complexes, which are required for pre-peptide maturation, are associated to the membrane by interaction with ABC transporters linked to the enzyme gene clusters (Siegers et al., 1996; Kiesau et al., 1997). More recently, the NRPS/PKS enzymes responsible for the biosynthesis of the antibiotic bacillaene and the siderophore pyoverdine were found to form membrane-associated mega complexes (Straight et al., 2007; Imperi and Visca, 2013). Presuming that CesCD is a membrane-bound protein, it could be involved in membrane-anchoring of the cereulide synthetase complex or other cereulide-processing enzymes, which otherwise may be non-functional. So far, immunoblot analysis using our anti-CesB antibody detected the cereulide synthetase only in cytosolic but not in membrane cell fractions (Rütschle, unpublished data). Therefore, further experiments targeting the localisation of cereulide and its synthetase complex are in progress to define the exact function of CesCD in cereulide synthesis.

## Conclusion

**FIGURE 6:**
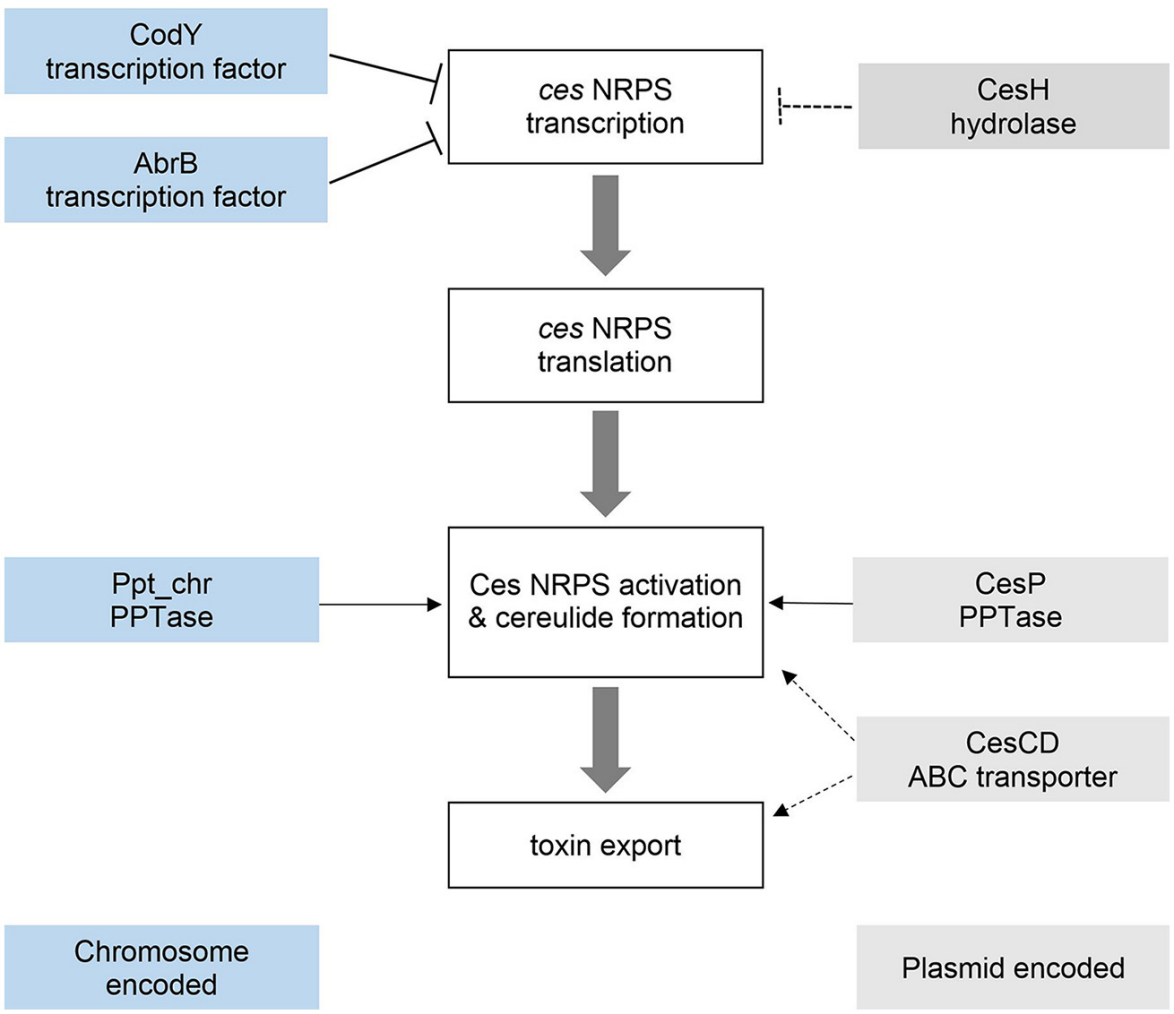
**Regulation of cereulide synthesis.** Interplay of chromosomally and plasmid encoded factors controlling cereulide synthesis at the transcriptional (AbrB, CodY, CesH) and post-translational (CesP, Ppt, cesCD) level.

Taken together, our data demonstrate the importance of the *cesAB*- adjacent genes *cesH, cesP*, and *cesCD* in the regulation of cereulide biosynthesis. Interestingly, these genes exert their regulatory functions on very different levels of toxin production, ranging from the transcriptional to the post-translational level (**Figure 6**). To our knowledge this is the first report on the impact of genes located within an NRPS encoding operon, and their gene products, on the transcription, translation and synthesis of the NRPS product and its biosynthetic machinery. The fact that not only chromosomal encoded master transcriptional regulators, such as AbrB or CodY, but also genes embedded in the *ces* gene cluster itself, influence cereulide production, highlights the complexity of regulation of synthesis of NRPS products.

## Conflict of Interest Statement

The authors declare that the research was conducted in the absence of any commercial or financial relationships that could be construed as a potential conflict of interest.

## Footnotes

1 http://blast.ncbi.nlm.nih.gov

2 http://www.cbs.dtu.dk/services/TMHMM/

3 http://www.ch.embnet.org/software/TMPRED_form.html

## References

[B1] Agata N. Ohta M. Mori M. Isobe M. (1995). A novel dodecadepsipeptide, cereulide, is an emetic toxin of *Bacillus cereus*. *FEMS Microbiol. Lett.* 129 17–20. 10.1111/j.1574-6968.1995.tb07550.x 7781985

[B2] Alonzo D. A. Magarvey N. A. Schmeing T. M. (2015). Characterization of cereulide synthetase, a toxin-producing macromolecular machine. *PLoS ONE* 10:e0128569 10.1371/journal.pone.0128569 PMC4455996 26042597

[B3] Bauer T. Stark T. Hofmann T. Ehling-Schulz M. (2010). Development of a stable isotope dilution analysis for the quantification of the *Bacillus cereus* toxin cereulide in foods. *J. Agric. Food Chem.* 58 1420–1428. 10.1021/jf9033046 19994891

[B4] Carlin F. Fricker M. Pielaat A. Heisterkamp S. Shaheen R. Salonen M. S. (2006). Emetic toxin-producing strains of *Bacillus cereus* show distinct characteristics within the *Bacillus cereus* group. *Int. J. Food Microbiol.* 109 132–138. 10.1016/j.ijfoodmicro.2006.01.022 16503068

[B5] Copp J. N. Neilan B. A. (2006). The phosphopantetheinyl transferase superfamily: phylogenetic analysis and functional implications in cyanobacteria. *Appl. Environ. Microbiol.* 72 2298–2305. 10.1128/AEM.72.4.2298-2305.2006 16597923 PMC1449050

[B6] Dassa E. Bouige P. (2001). The ABC of ABCS: a phylogenetic and functional classification of ABC systems in living organisms. *Res. Microbiol.* 152 211–229. 10.1016/S0923-2508(01)01194-9 11421270

[B7] Davidson A. L. Dassa E. Orelle C. Chen J. (2008). Structure, function, and evolution of bacterial ATP-binding cassette systems. *Microbiol. Mol. Biol. Rev.* 72 317–364. 10.1128/MMBR.00031-07 PMC2415747 18535149

[B8] Dierick K. Van Coillie E. Swiecicka I. Meyfroidt G. Devlieger H. Meulemans A. (2005). Fatal family outbreak of *Bacillus cereus*-associated food poisoning. *J. Clin. Microbiol.* 43 4277–4279. 10.1128/JCM.43.8.4277-4279.2005 16082000 PMC1233987

[B9] Dommel M. K. Frenzel E. Strasser B. Blöchinger C. Scherer S. Ehling-Schulz M. (2010). Identification of the main promoter directing cereulide biosynthesis in emetic *Bacillus cereus* and its application for real-time monitoring of ces gene expression in foods. *Appl. Environ. Microbiol.* 76 1232–1240. 10.1128/AEM.02317-09 20038713 PMC2820966

[B10] Dommel M. K. Lücking G. Scherer S. Ehling-Schulz M. (2011). Transcriptional kinetic analyses of cereulide synthetase genes with respect to growth, sporulation and emetic toxin production in *Bacillus cereus*. *Food Microbiol.* 28 284–290. 10.1016/j.fm.2010.07.001 21315985

[B11] Ehling-Schulz M. Frenzel E. Gohar M. (2015). Food – bacteria interplay: pathometabolism of emetic *Bacillus cereus*. *Front. Microbiol.* 6:704 10.3389/fmicb.2015.00704 PMC4500953 26236290

[B12] Ehling-Schulz M. Fricker M. Grallert H. Rieck P. Wagner M. Scherer S. (2006). Cereulide synthetase gene cluster from emetic *Bacillus cereus*: structure and location on a mega virulence plasmid related to *Bacillus anthracis* toxin plasmid pXO1. *BMC Microbiol.* 6:20 10.1186/1471-2180-6-20 PMC1459170 16512902

[B13] Ehling-Schulz M. Fricker M. Scherer S. (2004). *Bacillus cereus*, the causative agent of an emetic type of food-borne illness. *Mol. Nutr. Food Res.* 48 479–487. 10.1002/mnfr.200400055 15538709

[B14] Ehling-Schulz M. Svensson B. Guinebretiere M. H. Lindback T. Andersson M. Schulz A. (2005a). Emetic toxin formation of *Bacillus cereus* is restricted to a single evolutionary lineage of closely related strains. *Microbiology* 151 183–197. 10.1099/mic.0.27607-0 15632437

[B15] Ehling-Schulz M. Vukov N. Schulz A. Shaheen R. Andersson M. Martlbauer E. (2005b). Identification and partial characterization of the nonribosomal peptide synthetase gene responsible for cereulide production in emetic *Bacillus cereus*. *Appl. Environ. Microbiol.* 71 105–113. 10.1128/AEM.71.1.105-113.2005 15640177 PMC544239

[B16] Espinasse S. Gohar M. Lereclus D. Sanchis V. (2002). An ABC transporter from *Bacillus thuringiensis* is essential for beta-exotoxin I production. *J. Bacteriol.* 184 5848–5854. 10.1128/JB.184.21.5848-5854.2002 12374817 PMC135382

[B17] Finking R. Marahiel M. A. (2004). Biosynthesis of nonribosomal peptides1. *Annu. Rev. Microbiol.* 58 453–488. 10.1146/annurev.micro.58.030603.123615 15487945

[B18] Frenzel E. Doll V. Pauthner M. Lücking G. Scherer S. Ehling-Schulz M. (2012). CodY orchestrates the expression of virulence determinants in emetic *Bacillus cereus* by impacting key regulatory circuits. *Mol. Microbiol.* 85 67–88. 10.1111/j.1365-2958.2012.08090.x 22571587

[B19] Gebhard S. (2012). ABC transporters of antimicrobial peptides in Firmicutes bacteria – phylogeny, function and regulation. *Mol. Microbiol.* 86 1295–1317. 10.1111/mmi.12078 23106164

[B20] Hotelier T. Renault L. Cousin X. Negre V. Marchot P. Chatonnet A. (2004). ESTHER, the database of the alpha/beta-hydrolase fold superfamily of proteins. *Nucleic Acids Res.* 32 D145–D147. 10.1093/nar/gkh141 14681380 PMC308875

[B21] Imperi F. Visca P. (2013). Subcellular localization of the pyoverdine biogenesis machinery of *Pseudomonas aeruginosa*: a membrane-associated “siderosome”. *FEBS Lett.* 587 3387–3391. 10.1016/j.febslet.2013.08.039 24042050

[B22] Ivanova N. Sorokin A. Anderson I. Galleron N. Candelon B. Kapatral V. (2003). Genome sequence of *Bacillus cereus* and comparative analysis with *Bacillus anthracis*. *Nature* 423 87–91. 10.1038/nature01582 12721630

[B23] Kiesau P. Eikmanns U. Gutowski-Eckel Z. Weber S. Hammelmann M. Entian K. D. (1997). Evidence for a multimeric subtilin synthetase complex. *J. Bacteriol.* 179 1475–1481. 9045802 10.1128/jb.179.5.1475-1481.1997 PMC178855

[B24] Lambalot R. H. Gehring A. M. Flugel R. S. Zuber P. Lacelle M. Marahiel M. A. (1996). A new enzyme superfamily – the phosphopantetheinyl transferases. *Chem. Biol.* 3 923–936. 10.1016/S1074-5521(96)90181-7 8939709

[B25] Lane A. L. Moore B. S. (2011). A sea of biosynthesis: marine natural products meet the molecular age. *Nat. Prod. Rep.* 28 411–428. 10.1039/c0np90032j 21170424 PMC3101795

[B26] Letunic I. Doerks T. Bork P. (2012). SMART 7: recent updates to the protein domain annotation resource. *Nucleic Acids Res.* 40 D302–D305. 10.1093/nar/gkr931 22053084 PMC3245027

[B27] Linton K. J. Cooper H. N. Hunter I. S. Leadlay P. F. (1994). An ABC-transporter from *Streptomyces longisporoflavus* confers resistance to the polyether-ionophore antibiotic tetronasin. *Mol. Microbiol.* 11 777–785. 10.1111/j.1365-2958.1994.tb00355.x 8196549

[B28] Lücking G. Dommel M. K. Scherer S. Fouet A. Ehling-Schulz M. (2009). Cereulide synthesis in emetic *Bacillus cereus* is controlled by the transition state regulator AbrB, but not by the virulence regulator PlcR. *Microbiology* 155 922–931. 10.1099/mic.0.024125-0 19246763

[B29] Magarvey N. A. Ehling-Schulz M. Walsh C. T. (2006). Characterization of the cereulide NRPS alpha-hydroxy acid specifying modules: activation of alpha-keto acids and chiral reduction on the assembly line. *J. Am. Chem. Soc.* 128 10698–10699. 10.1021/ja0640187 16910662

[B30] Marahiel M. A. Stachelhaus T. Mootz H. D. (1997). Modular peptide synthetases involved in nonribosomal peptide synthesis. *Chem. Rev.* 97 2651–2674. 10.1021/cr960029e 11851476

[B31] Marxen S. Stark T. D. Frenzel E. Rütschle A. Lücking G. Purstinger G. (2015a). Chemodiversity of cereulide, the emetic toxin of *Bacillus cereus*. *Anal. Bioanal. Chem.* 407 2439–2453. 10.1007/s00216-015-8511-y 25665710

[B32] Marxen S. Stark T. D. Rütschle A. Lücking G. Frenzel E. Scherer S. (2015b). Depsipeptide Intermediates Interrogate Proposed Biosynthesis of Cereulide, the Emetic Toxin of *Bacillus cereus*. *Sci. Rep.* 5 10637 10.1038/srep10637 PMC4445039 26013201

[B33] May J. J. Wendrich T. M. Marahiel M. A. (2001). The dhb operon of *Bacillus subtilis* encodes the biosynthetic template for the catecholic siderophore 2,3-dihydroxybenzoate-glycine-threonine trimeric ester bacillibactin. *J. Biol. Chem.* 276 7209–7217. 10.1074/jbc.M009140200 11112781

[B34] McMorran B. J. Merriman M. E. Rombel I. T. Lamont I. L. (1996). Characterisation of the pvdE gene which is required for pyoverdine synthesis in *Pseudomonas aeruginosa*. *Gene* 176 55–59. 10.1016/0378-1119(96)00209-0 8918232

[B35] Mesnage S. Fontaine T. Mignot T. Delepierre M. Mock M. Fouet A. (2000). Bacterial SLH domain proteins are non-covalently anchored to the cell surface via a conserved mechanism involving wall polysaccharide pyruvylation. *EMBO J.* 19 4473–4484. 10.1093/emboj/19.17.4473 10970841 PMC302060

[B36] Messelhäusser U. Frenzel E. Blöchinger C. Zucker R. Kämpf P. Ehling-Schulz M. (2014). Emetic *Bacillus cereus* are more volatile than thought: recent foodborne outbreaks and prevalence studies in Bavaria (2007-2013). *Biomed Res. Int.* 2014 465603 10.1155/2014/465603 PMC4033357 24895578

[B37] Mootz H. D. Marahiel M. A. (1997). The tyrocidine biosynthesis operon of *Bacillus brevis*: complete nucleotide sequence and biochemical characterization of functional internal adenylation domains. *J. Bacteriol.* 179 6843–6850. 9352938 10.1128/jb.179.21.6843-6850.1997 PMC179617

[B38] Murphy E. (1985). Nucleotide sequence of a spectinomycin adenyltransferase AAD(9) determinant from *Staphylococcus aureus* and its relationship to AAD(3”) (9). *Mol. Gen. Genet.* 200 33–39. 10.1007/BF00383309 2993813

[B39] Nakano M. M. Corbell N. Besson J. Zuber P. (1992). Isolation and characterization of sfp: a gene that functions in the production of the lipopeptide biosurfactant, surfactin, in *Bacillus subtilis*. *Mol. Gen. Genet.* 232 313–321. 1557038 10.1007/BF00280011

[B40] Naranjo M. Denayer S. Botteldoorn N. Delbrassinne L. Veys J. Waegenaere J. (2011). Sudden death of a young adult associated with *Bacillus cereus* food poisoning. *J. Clin. Microbiol.* 49 4379–4381. 10.1128/JCM.05129-11 22012017 PMC3232990

[B41] Pezard C. Berche P. Mock M. (1991). Contribution of individual toxin components to virulence of *Bacillus anthracis*. *Infect. Immun.* 59 3472–3477. 1910002 10.1128/iai.59.10.3472-3477.1991 PMC258908

[B42] Pfaﬄ M. W. Horgan G. W. Dempfle L. (2002). Relative expression software tool (REST) for group-wise comparison and statistical analysis of relative expression results in real-time PCR. *Nucleic Acids Res.* 30 e36 10.1093/nar/30.9.e36 PMC113859 11972351

[B43] Podlesek Z. Comino A. Herzog-Velikonja B. Zgur-Bertok D. Komel R. Grabnar M. (1995). *Bacillus licheniformis* bacitracin-resistance ABC transporter: relationship to mammalian multidrug resistance. *Mol. Microbiol.* 16 969–976. 10.1111/j.1365-2958.1995.tb02322.x 7476193

[B44] Quigley N. B. Mo Y. Y. Gross D. C. (1993). SyrD is required for syringomycin production by *Pseudomonas* syringae pathovar syringae and is related to a family of ATP-binding secretion proteins. *Mol. Microbiol.* 9 787–801. 10.1111/j.1365-2958.1993.tb01738.x 8231810

[B45] Rasko D. A. Ravel J. Okstad O. A. Helgason E. Cer R. Z. Jiang L. (2004). The genome sequence of *Bacillus cereus* ATCC 10987 reveals metabolic adaptations and a large plasmid related to *Bacillus anthracis* pXO1. *Nucleic Acids Res.* 32 977–988. 10.1093/nar/gkh258 14960714 PMC373394

[B46] Schwarzer D. Mootz H. D. Linne U. Marahiel M. A. (2002). Regeneration of misprimed nonribosomal peptide synthetases by type II thioesterases. *Proc. Natl. Acad. Sci. U.S.A.* 99 14083–14088. 10.1073/pnas.212382199 12384573 PMC137840

[B47] Siegers K. Heinzmann S. Entian K. D. (1996). Biosynthesis of lantibiotic nisin. Posttranslational modification of its prepeptide occurs at a multimeric membrane-associated lanthionine synthetase complex. *J. Biol. Chem.* 271 12294–12301. 10.1074/jbc.271.21.12294 8647829

[B48] Spizizen J. (1958). Transformation of biochemically deficient strains of *Bacillus Subtilis* by deoxyribonucleate. *Proc. Natl. Acad. Sci. U.S.A.* 44 1072–1078. 10.1073/pnas.44.10.1072 16590310 PMC528696

[B49] Stark T. Marxen S. Ruetschle A. Luecking G. Scherer S. Ehling-Schulz M. (2013). Mass spectrometric profiling of *Bacillus cereus* strains and quantitation of the emetic toxin cereulide by means of stable isotope dilution analysis and HEp-2 bioassay. *Anal. Bioanal. Chem.* 405 191–201. 10.1007/s00216-012-6485-6 23079954

[B50] Straight P. D. Fischbach M. A. Walsh C. T. Rudner D. Z. Kolter R. (2007). A singular enzymatic megacomplex from *Bacillus subtilis*. *Proc. Natl. Acad. Sci. U.S.A.* 104 305–310. 10.1073/pnas.0609073103 17190806 PMC1765455

[B51] Tamura K. Peterson D. Peterson N. Stecher G. Nei M. Kumar S. (2011). MEGA5: molecular evolutionary genetics analysis using maximum likelihood, evolutionary distance, and maximum parsimony methods. *Mol. Biol. Evol.* 28 2731–2739. 10.1093/molbev/msr121 21546353 PMC3203626

[B52] Thompson J. D. Higgins D. G. Gibson T. J. (1994). CLUSTAL W: improving the sensitivity of progressive multiple sequence alignment through sequence weighting, position-specific gap penalties and weight matrix choice. *Nucleic Acids Res.* 22 4673–4680. 10.1093/nar/22.22.4673 7984417 PMC308517

[B53] Trieu-Cuot P. Carlier C. Martin P. Courvalin P. (1987). Plasmid transfer by conjugation from *Escherichia coli* to Gram-positive bacteria. *FEMS Microbiol. Lett.* 48 289–294. 10.1111/j.1574-6968.1987.tb02558.x

[B54] Tschiedel E. Rath P. M. Steinmann J. Becker H. Dietrich R. Paul A. (2015). Lifesaving liver transplantation for multi-organ failure caused by *Bacillus cereus* food poisoning. *Pediatr. Transplant.* 19 E11–E14. 10.1111/petr.12378 25323120

[B55] Walsh C. T. Gehring A. M. Weinreb P. H. Quadri L. E. Flugel R. S. (1997). Post-translational modification of polyketide and nonribosomal peptide synthases. *Curr. Opin. Chem. Biol.* 1 309–315. 10.1016/S1367-5931(97)80067-1 9667867

[B56] Wenzel S. C. Müller R. (2005). Formation of novel secondary metabolites by bacterial multimodular assembly lines: deviations from textbook biosynthetic logic. *Curr. Opin. Chem. Biol.* 9 447–458. 10.1016/j.cbpa.2005.08.001 16107321

[B57] Yakimov M. M. Kroger A. Slepak T. N. Giuliano L. Timmis K. N. Golyshin P. N. (1998). A putative lichenysin A synthetase operon in *Bacillus licheniformis*: initial characterization. *Biochim. Biophys. Acta* 1399 141–153. 10.1016/S0167-4781(98)00096-7 9765590

