# Supplementary material for: Ces locus embedded proteins control the non-ribosomal synthesis of the cereulide toxin in emetic Bacillus cereus on multiple levels
Source: Front Microbiol. 2015 Oct 13;6:1101. doi: 10.3389/fmicb.2015.01101 (PMC4602138; doi:10.3389/fmicb.2015.01101)
Supplement: Supplementary file 1 [file Data_Sheet_1.PDF]

Supplementary Material

Ces locus embedded proteins control the nonribosomal synthesis of the cereulide toxin in emetic *Bacillus cereus* on multiple levels

Genia Lücking[1], Elrike Frenzel[1,2,4#], Andrea Rütschle[1#], Sandra Marxen[3], Timo D. Stark[3], Thomas Hofmann[3], Siegfried Scherer[1,5], Monika Ehling-Schulz[2*]

*** Correspondence:** Monika.Ehling-Schulz@vetmeduni.ac.at

1. Supplementary Figures

FIGURE S1

```
                                                         Walker A
CesC            MIKDVGKMIKIEGLTKEFGNGKGIFDVTFEVKQGEVFGFLGPNGAGKSTTIRHLMGFIKP 60
BerA            -------MISVQNVTKQFSNGKGLFHITFNVKEGEVFGYLGPNGAGKSTTIRNLMGFIKP 53
TnrB2           ----------MAGLHKSFGRTHALDGLDLAVDSGEVHGFLGPNGAGKSTTIRVLLGLLRA 50
BcrA            ----MSTIIKTTDLTKMYGSQKSVDHLNINVKQGDIYGFLGRNGAGKTTTIRMLLGLIKP 56
                             : * :   : :  : : *  *:: *:** *****:**** *:*:::


CesC            SKGAATINGFDCWKEADQVKKHVGYLPGEIAFPEEMTAYDFIRLIAGMKGMSDLGRAEEF 120
BerA            TSGKSSIFGLDCWNEAAKIQREVGYLPGEISFIEGMNGLEFLKLMQGMRGLKDTKRRDEL 113
TnrB2           DSGAPSSSARTPWKDAVALHRRLAYVPGDVELWPNLTGGEAIDLLSRLRGGLDRQRRDEL 110
BcrA            TSGQIEIFGENFFKNKKEILRRIGSIVEVPGFYANLTARENLLINAKIIGIHKKNAIDEV 116
                  *         :::   : : :  :     :   :   : : :   : *       :*


                                  Linker                 Walker B
CesC            IEKFQLDMRVH--IKKMSKGMKQKLAIVAAFLHDPDILILDEPSTGLDPLMQNILVDFIL 178...298
BerA            IERLQFDVKTP--IRKMSKGMKQKVGIVAAFMHDPEVLILDEPTSGLDPLMQQVFIDLIV 171...297
TnrB2           IERFDLDPTKK--GRAYSKGNRQKVPIVAALASDAELLLLDEPTAGLDPLMEVVFQDVIL 168...300
BcrA            LEIVGLQHETKKLVGKFSLGMKQRLGIARALLHYPELLILDEPTNGLDPIGIKEMRRLIH 176...306
                :*   ::          * * :*:: *  *:    ::*:****: ****:    :   *
```

Supplementary Figure S1: Sequence alignment of CesC with selected homologous ATP-binding proteins: BerA of *B. thuringiensis* (50% identity), TnrB2 of *Streptomyces longisporoflavus* (33% identity) and BcrA of *B. licheniformis* (32% identity). Conserved nucleotide-binding motifs are highlighted in gray, identical residues with asterisk, similar residues with colon.

FIGURE S2

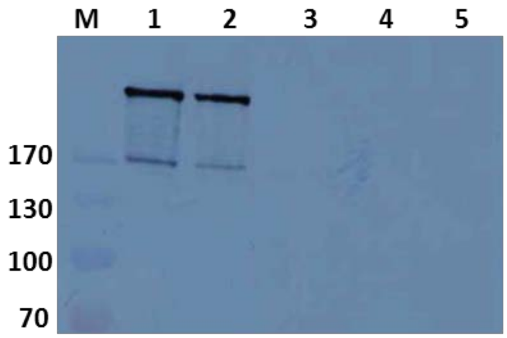

Supplementary Figure S2: Western blot analysis of CesB expression using a monoclonal anti-CesB antibody. Identical amounts (30 µg) of total protein from various *B. cereus* und *B. subtilis* strains, harvested at exponential growth phase (OD_{600} = 8), were separated by SDS-PAGE. Lanes: M: protein ladder 70-170 kDa; 1: emetic *B. cereus* F4810/72; 2: emetic *B. cereus* A529 3: non-emetic *B. cereus* ATCC10987; 4: non-emetic *B. cereus* ATCC14579; 5: *B. subtilis* 168

FIGURE S3

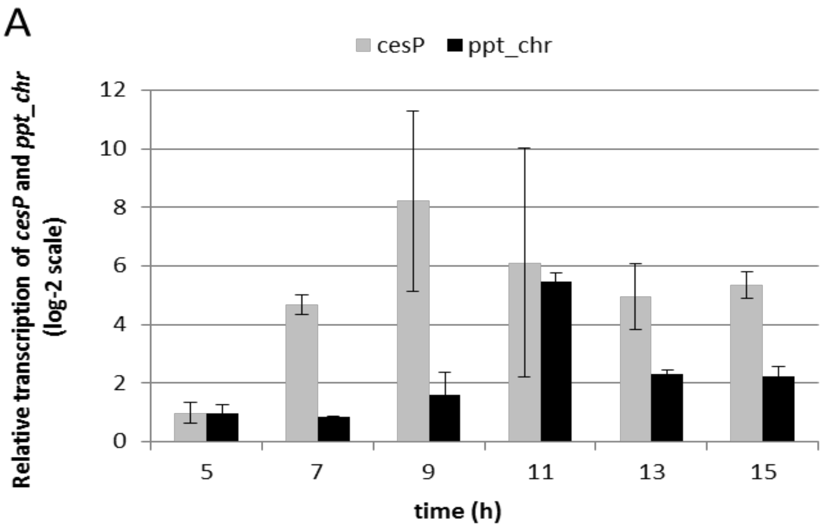

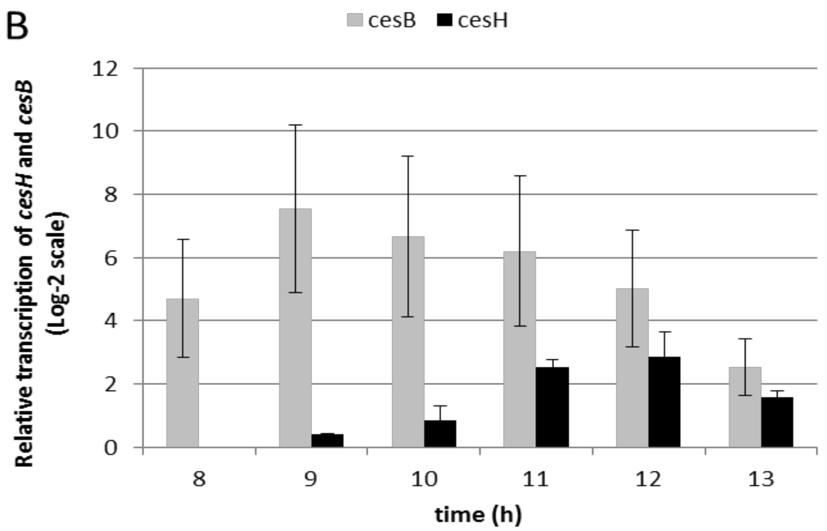

Supplementary Figure S3: Comparative transcription kinetics of *cesP / ppt* (A) and *cesB / cesH* (B) of *B. cereus* F4810/72 during growth in LB media. Transcript levels were quantified by RT-qPCR relatively to 16 *rrn* levels as described in material and methods.

2. Supplementary Tables

TABLE S1: Plasmids used in this study.

Plasmids	Relevant characteristics	Reference
pMAD	thermosensitive shuttle vector for Gram-positive bacteria; Amp^r, Ery^r	(Arnaud et al., 2004)
pMAD/cesH	pMAD derivative containing a 293 bp inner fragment of *cesH*; Amp^r, Ery^r	This study
pMAD/ppt	pMAD derivative containing a 327 bp inner fragment of *ppt* (ACJ79141); Amp^r, Ery^r	This study
pAD123	shuttle vector for Gram positive hosts, containing a promoter-less *gfp*mut3a; Amp^r, Cm^r	(Dunn and Handelsman, 1999)
pAD/Pro-ces/cesCD	pAD123 derivative containing the regulatory *ces* promoter region (~500bp) in front of *cesCD* (*gfp*-less); Amp^r, Cm^r	This study
pAD/Pro-ces/cesP	pAD123 derivative containing the regulatory *ces* promoter region (~500bp) in front of *cesP* (*gfp*-less); Amp^r, Cm^r	This study
pMM1522	Shuttle and *Bacillus* expression vector with xylose- inducible promoter; Amp^r, Tc^r	MoBiTec
pMM/ppt	pMM1522 derivate containing promoter-less *ppt;* xylose- inducible; Amp^r, Tc^r	This study
pMM/cesH	pMM1522 derivate containing promoter-less *cesH;* xylose- inducible; Amp^r, Tc^r	This study
TOPO pCR 2.1	cloning vector ; Amp^r, Kan^r	Invitrogen
pUC1318spc	pUC1318 carrying the *spc* spectinomycin resistance gene; Amp^r, Spc^r	(Murphy, 1985;Mesnage et al., 2000)
pAT113	conjugative suicide vector for *Bacillus*; Kan^r, Ery^r	(Trieu-Cuot et al., 1991)
pSPCH+2	pUC19 carrying a non-polar mutagenic Spc	(Mesnage et al.,

	resistence cassette; Amp^r, Spc^r	2000)
pAT113△cesP/spc	pAT113 derivative containing the up- and downstream regions of *cesP* flanking the Spc resistance cassette from pUC1318spc; Spc^r, Ery^r, Kan^r	This study
pAT113△cesP/spcH+2	pAT113 derivative containing the up- and downstream regions of *cesP* flanking a non-polar Spc resistance cassette from pSPCH+2; Spc^r, Ery^r, Kan^r	This study
pAT113△cesCD/spc	pAT113 derivative containing the up- and downstream regions of *cesCD* flanking the Spc resistance cassette from pUC1318spc; Spc^r, Ery^r, Kan^r	This study
pET28b(+)	*E. coli* expression vector; T7*lac* promoter, His_6 tag; Kan^r	Novagen
pET28-cesB1	CesB1 module of the cereulide synthetase including its predicted ribosome binding site with N-terminal His_6 tag in pET28b; Kan^r	This study
pWH1520	*Bacillus* expression vector with xylose inducible promoter; Amp^r, Tc^r	(Rygus and Hillen, 1991)
pWHCesB1His	CesB1 module of the cereulide synthetase including its predicted ribosome binding site with N-terminal His_6 tag in pWH1520; Amp^r, Tc^r	This study

TABLE S2: Oligonucleotides used for cloning and RT-qPCR.

Primer	Sequence (5' – 3')[a]	Target	Usage
16SrRNA1	GGAGGAAGGTGGGGATGACG	241 bp inner fragment of *rrn*; RT-qPCR	RT-qPCR
16SrRNA2	ATGGTGTGACGGGCGGTGTG		
cesA_for	GATTACGTTCGATTATTTGAAG	197 bp inner fragment of *cesA*; RT-qPCR	RT-qPCR
cesA_rev	CGTAGTGGCAATTTCGCAT		

cesP_up_F<br>cesP_up_R	TTTGAGCTCTTTAGTTCCTTCATGAC<br>TTCACCCGGGTAAATCTTTATGGAAT	~1 kb upstream fragment of *cesP* including 38 bp of the 5'end of *cesP*	deletion mutant
cesP_down_F<br>cesP_down_R	TCATCCCGGGTATAAAGTCGCGGTTT<br>TCAAGAGCTCCATCAACATAGTCATA	~1 kb downstream fragment of *cesP* including 153 bp of the 3'end of *cesP*	deletion mutant
cesCD_up_F<br>cesCD_up_R	ATTGAATTCAAATGCGTGAAGGTAGTGA<br>AATCCCGGGAAATGTCGAATTGTTGTGG	~1,1 kb upstream fragment of *cesC* including 140 bp of the 5'end of *cesC*	deletion mutant
cesCD_down_F<br>cesCD_down_R	TTGCCCGGGTTGCGAATACAGAAAGCTTAG<br>TTTGAATTCCTCTACTGTTGGTACTAGTTG	~1,2 kb downstream fragment of *cesD* including 257 bp of the 3' end of *cesD*	deletion mutant
cesCD_F_Xba<br>cesCD_R_Pae	TGGTCTAGAAGCATATTTAGAGTTG<br>TTTGCATGCTATTTAAACATCTTAT	~1,8 kb fragment containing the genes *cesC* and *cesD*	complementation mutant
cesP_Pro_F2<br>cesP_Pro_R	TATGAATTCCCTCTTGGCTTTCGGCTACGAT<br>ATAGCATGCACGTTTAATTCAGGACTAGCTCTC	~1,2 kb fragment containing the *cesP* gene with the preceding *ces* promoter region	complementation mutant
ppt_chr_F<br>ppt_chr_R	GCGGAATTCAAGAAGTTGTAAATGAATAGGTTTG<br>AATGCATGCTGAGGAACACCTCTTCTATAAAATA	~900 bp fragment containing the *ppt* gene (ACJ79141) (promoterless)	complementation mutant
cesH_F_speI<br>cesH_R_sphI	ATAACTAGTGGGGATTGCTTGTATTA<br>AAGGCATGCAGGAACTAAAGAG	~800 bp fragment containing the *cesH* gene	over-expression

		(promoterless)	mutant
cesCD_F2_speI<br>cesCD_R_sphI	TATACTAGTTTGATAAAGGATGTGGG<br>TTAGCATGCTTATAAGTTCAAATCTTTAT	~1,8 kb fragment containing the *cesCD* genes (promoterless)	over-expression mutant
cesH_F_300<br>cesH_R_300	TCAAAGCTTAGTTCTTGACCTAC<br>TTTGAATTCCCAGTGGTAATGCC	293 bp inner fragment of *cesH*	insertion mutant
ppt_F_300<br>ppt_R_300	TCAGAATTCGGCAGATCGTGCTCG<br>TTAGGATCCTTCATAACTTGCGC	327 bp inner fragment of *ppt* (locus tag BCAH187_A2475)	insertion mutant
cesB135Xho_rev<br>cesB135Nco_for	AGGGAGCTCAGGCATTCTCACTCGGCAA A<br>GGCCATGGGGACTGAAGCAGCAACGAT T	~4 kp fragment of *cesB* including the ribosome binding site	CesB over-expression and purification
cesBpETSpe_for2<br>cesBpETSph_rev2	CCGACTAGTGCGGATAACAATTCCCCTC<br>TAGCATGCCGGATATAGTTCCTCCTTTCA	~4 kp fragment of *cesB* including the ribosome binding site	CesB over-expression and purification
